# Machine learning-based detection and mapping of riverine litter utilizing Sentinel-2 imagery

**DOI:** 10.1007/s11356-023-27068-0

**Published:** 2023-04-28

**Authors:** Ahmed Mohsen, Tímea Kiss, Ferenc Kovács

**Affiliations:** 1 Department of Geoinformatics, Physical and Environmental Geography, University of Szeged, Egyetem u. 2–6, Szeged, 6722 Hungary; 2 Department of Irrigation and Hydraulics Engineering, Tanta University, Tanta, 31512 Egypt

**Keywords:** Tisza River, Plastic indices, Litter transport, Support vector classifier, Artificial neural network, Macroplastic

## Abstract

Despite the substantial impact of rivers on the global marine litter problem, riverine litter has been accorded inadequate consideration. Therefore, our objective was to detect riverine litter by utilizing middle-scale multispectral satellite images and machine learning (ML), with the Tisza River (Hungary) as a study area. The Very High Resolution (VHR) images obtained from the Google Earth database were employed to recognize some riverine litter spots (a blend of anthropogenic and natural substances). These litter spots served as the basis for training and validating five supervised machine-learning algorithms based on Sentinel-2 images [Artificial Neural Network (ANN), Support Vector Classifier (SVC), Random Forest (RF), Naïve Bays (NB) and Decision Tree (DT)]. To evaluate the generalization capability of the developed models, they were tested on larger unseen data under varying hydrological conditions and with different litter sizes. Besides the best-performing model was used to investigate the spatio-temporal variations of riverine litter in the Middel Tisza. According to the results, almost all the developed models showed favorable metrics based on the validation dataset (e.g., F1-score; SVC: 0.94, ANN: 0.93, RF: 0.91, DT: 0.90, and NB: 0.83); however, during the testing process, they showed medium (e.g., F1-score; RF:0.69, SVC: 0.62; ANN: 0.62) to poor performance (e.g., F1-score; NB: 0.48; DT: 0.45). The capability of all models to detect litter was bounded to the pixel size of the Sentinel-2 images. Based on the spatio-temporal investigation, hydraulic structures (e.g., Kisköre Dam) are the greatest litter accumulation spots. Although the highest transport rate of litter occurs during floods, the largest litter spot area upstream of the Kisköre Dam was observed at low stages in summer. This study represents a preliminary step in the automatic detection of riverine litter; therefore, additional research incorporating a larger dataset with more representative small litter spots, as well as finer spatial resolution images is necessary.

## Introduction

The rivers are the main source of marine litter, as approximately 80% of marine litter originates from inland sources (González et al. 2021, González et al. 2016). The term “riverine litter” refers to the natural and artificial materials transported by a river, which are often trapped by the banks and hydraulic structures. The natural and artificial litter is mixed and drifting together in a form of litter spots. The litter can enter the fluvial system by run-off and direct input, and during its transportation, it degrades to smaller particles (González et al. 2016). The artificial floating materials, especially plastics, originate from public waste, landfill sites, agricultural fields, and industrial disposals (Van Emmerik et al. 2018). Rech et al. (2014) classified artificial litter according to its ability to float, referring to persistent buoyant (plastics and wood), short-time buoyant (cigarette stubs, paper, and textiles), and non-buoyant litter (concrete, metal, and glass).

The monitoring of riverine litter aims to identify its sources, quantity, and fluxes, which aids researchers and decision-makers in evaluating their environmental impacts. Sampling locations usually are at potential litter hotspots, which are close to populated areas. The sampling timing is influenced by hydro-meteorological conditions (e.g., rainfall, flood) and human factors (e.g., operation of dams). Until now, there is no worldwide standardized approach for riverine litter monitoring. Within the observation methods the quantity and litter type are visually determined (Doyle et al. 2007, Van Emmerik et al. 2018) or automatically with the aid of images collected by cameras (De Giglio et al. 2021, Kataoka &Nihei 2020), drones (Geraeds et al. 2019, Wolf et al. 2020) or satellites (Biermann et al. 2020, Jakovljević et al. 2019, Themistocleous et al. 2020). The collection methods depend on collecting litter using nets, sieves, or pumps, and the samples are analyzed in the field or laboratory.

The automatic monitoring of riverine litter enables continuous observation, resulting in more representative litter data. Though drones and fixed cameras provide very high-resolution images, they usually cover a limited area. Meanwhile, multispectral satellites provide coarser images, but they cover large areas. The current development in the spatial, spectral, and temporal resolutions of many earth observation systems (e.g., Landsat, Sentinel-2, Worldview, Spot) could be exploited to monitor riverine litter periodically. Therefore, this study focuses on the development of riverine litter detectors based on satellite images, which would provide more riverine litter data due to their wide coverage. Deriving a model to detect riverine litter is challenging, as the process is influenced by several factors related to water, atmosphere, sunlight, and the litter itself. Goddijn-Murphy et al. (2018) stated that the downwelling sunbeam behaves differently with the floating litter than with water in terms of reflectance, transmission into the water, and reflection passing through the litter, which paved the road of automatic detection of litter. However, the spatial and spectral resolutions of the employed sensor are still questionable.

Hu (2021) studied the extent to which the various forms of plastic litter could be detected by the visible and near-infrared spectral bands, concluding that microplastic detection is impossible by any present or planned optical sensors; however, macro-plastics are possible. Topouzelis et al. (2019) investigated the capability of various remote sensing platforms (i.e., UAV cameras, Sentinel-1, and Sentinel-2) to detect artificial plastic targets, and the results revealed the potential of these platforms to detect plastics; however, the detection influenced by the sub-pixel coverage of plastic. Martínez-Vicente et al. (2019) studied the prerequisites for a satellite sensor platform that can be used to detect marine plastic debris and concluded that the NIR and SWIR bands are the most suitable for this purpose.

The remote sensing methods used to monitor litter can be categorized into three groups. (1) Bio-optical modeling method involves using a model to predict the optical properties of water, including absorption and scattering of light, in the presence of litter. The model is highly influenced by the spectral signature of litter and water, as well as the geometrical optics of the sensor. (2) Deep learning models aim to automate the detection of litter through the application of computer vision tasks such as image classification (Jakovljević et al. 2019), object detection (Hegde et al. 2021), and image segmentation (Mifdal et al. 2021). (3) Indices method aims to develop an index that discriminates between litter and water based on their spectral signatures. For instance, the Floating Debris Index (FDI) (Biermann et al. 2020) was developed based on enhancing the difference between the near-infrared (NIR) band and the baseline reflectance of near-infrared. The Plastic Index (PI) (Themistocleous et al. 2020) detected macro-plastics in marine environments effectively by combining the visible (red) and near-infrared (NIR) bands.

Although bio-optical modelling could detect riverine litter with an elevated accuracy, few studies attempted to apply it due to its complexity and sensitivity to slight changes in the optical properties of water caused by changes in the concentrations of active water constituents (Moore et al. 2014). Goddijn-Murphy et al. (2018) proposed two structures of bio-optical models for detecting the fraction of the surface area of macro-plastic over the total surface area of water based on single and dual bands. The detection of the spectral indices usually needs further analysis since they usually mix between litter spots and other materials. For example, Biermann et al. (2020) improved FDI identifications by using the Normalized Difference Vegetation Index (NDVI) to eliminate false vegetation identifications and the Naive Bayes algorithm for separating different litter materials.

Therefore, this study focuses mainly on image classification employing some traditional machine learning algorithms to detect riverine litter due to its simplicity, high detection accuracy, and generalization capability (Szeliski 2010). De Giglio et al. (2021) applied supervised (ISODATA and K-means), unsupervised (Maximum likelihood), and machine learning (decision tree) classification techniques to detect plastic litter in images collected by the multispectral camera from four different environments in the Reno River, Italy. Unsupervised classifiers lost most plastic samples, while supervised ones only detected pure plastic and misclassified iron as plastic. The decision tree had the best accuracy (>80%). Other research has attempted to use deep learning algorithms for detecting litter; however, these algorithms require a large amount of training data which is not readily available in the case of riverine litter due to a shortage of data. In contrast, traditional machine learning algorithms can often achieve good performance with limited data (Shinde &Shah 2018). A novel Conventional Neural Network (CNN)-based models for detecting and quantifying litter and macro-plastics was developed by Wolf et al. (2020). It consists of two parts: Plastic Litter Detector (PLD)-CNN (83% accuracy) classifying images into categories (e.g., sand, water, plastic, and vegetation) and Plastic Litter Quantifier (PLQ)-CNN (71% accuracy) classifying macro-plastics into subcategories (e.g., bottles and bags). A recent study presented an automatic learning approach to detect riverine litter based on image segmentation architectures (e.g., U-Net and DeeplabV3+) with a mean Intersection over Union (IoU) of 0.82 (Solé Gómez et al. 2022). The dataset of litter spots was created based on news, social media, and published articles due to the shortage of riverine litter data.

Most of the studies were performed on seas (Biermann et al. 2020, Themistocleous et al. 2020), and very few in rivers (Jakovljević et al. 2019, Solé Gómez et al. 2022), thus more research efforts are needed to understand the sources, pathways, and sinks of riverine litter. The availability of litter data was a challenge for many studies; thus they constructed artificial litter targets (Themistocleous et al. 2020), or collect data on litter accumulation events from social media or scientific reports (Biermann et al. 2020, Solé Gómez et al. 2022). Furthermore, there is a lack of litter detector models with sufficient generalization capability (Jia et al. 2023). Therefore, we aimed to offer an alternative methodological approach based on very high spatial resolution images (i.e., Google Earth) as a source for riverine litter data to build riverine litter detectors based on Sentinel 2 images and machine learning (ML) algorithms and testing their generalization capabilities. Our goals are to (1) test high spatial resolution images as a source of riverine litter distribution data; (2) compare various ML algorithms (DT, NB, RF, SVM, and ANN) to detect riverine litter spots based on Sentinel-2 images; (3) define the areal limit of the litter spots that could be recognized by the Sentinel-2 images and testing their generalization capabilities; (4) reveal the spatio-temporal dynamism of the riverine litter along a 175 km-long reach of a medium-sized river to support mitigation measures.

## Study Area

**Figure 1.**
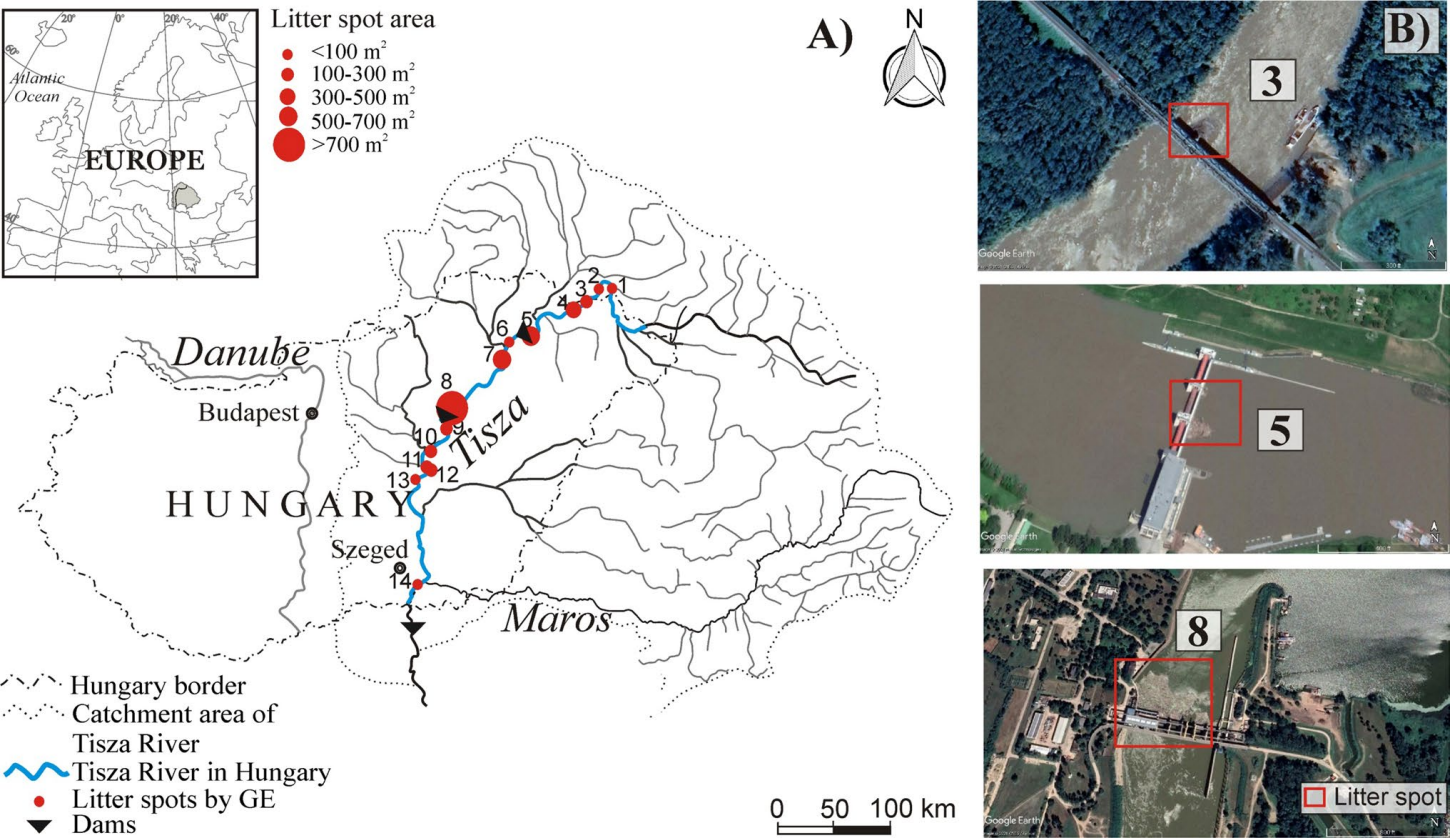
**A**) The Tisza River springs in Ukraine, joining the Danube River in Serbia. At some locations (1-14), litter spots were identified based on the Google Earth database. **B**) Examples of the identified litter spots on Very High Resolution (VHR) images. The numbers (3, 5, and 8) refer to the number of litter spots shown in A

The Tisza River is a main tributary of the Danube (catchment area: 157,200 km^2^, length: 966 km; ICPDR 2008). Its catchment area is shared between five countries (Romania: 46.2%, Hungary: 29.4%, Slovakia: 9.7%, Ukraine: 8.1%, and Serbia: 6.6%) (Figure 1), which causes environmental conflicts between the upstream and downstream countries. During floods, the river conveys a significant amount of sediment and litter due to the inundation of industrial, mining, agricultural, and municipal waste areas. Flood waves usually develop in early spring and early summer, whereas low stages are typical in the second half of the year (Amissah et al. 2018). The flow conditions, and thus the litter transport and accumulation, are influenced by three dams. The channel of the Tisza (mean width: 164 m) is sinuous with highly meandering sections, where the bank erosion rate is 1.7 m/y (Kiss et al. 2019), thus at these sections, organic debris frequently gets into the river. The mean channel depth is 14.1 m; thus, the woody debris is easily transported away and rarely stranded.

**Figure 2.**
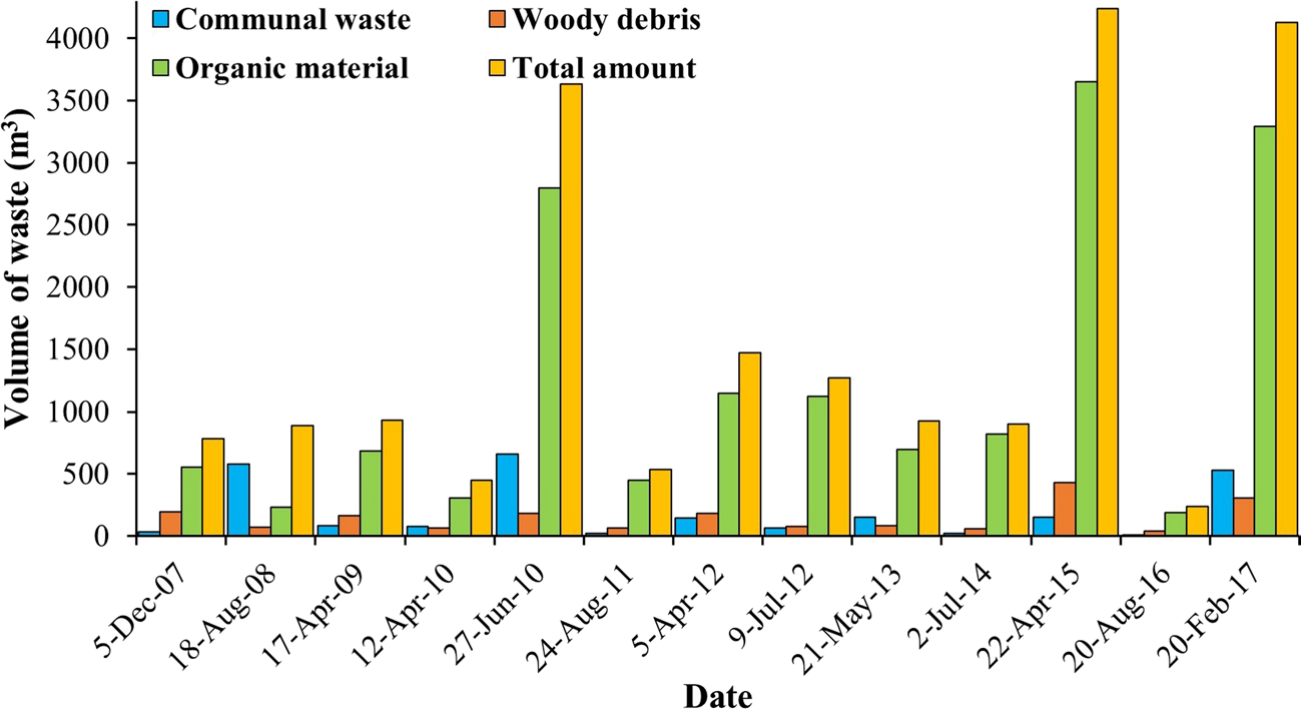
Temporal changes of the removed litter at the Kisköre Dam, Middle Tisza (2007-2017) (data source; Katona 2019)

The greatest waste producer in the catchment is Ukraine (12 million tons/year), while the least is produced in Slovakia (2.4 million tons/year). In Serbia and Ukraine, only ca. 1% of the waste is recycled, and the situation is best in Hungary (36%) and Slovakia (38%) (Cewep 2021). To gain an insight into the quantity of transported riverine litter in the Tisza, the Hungarian authorities remove 90 to 10,000 tons of floating litter annually from the river, most of which (ca. 67%) are from the Hungarian Upper Tisza (Idex.hu 2019, Siklósi 2017). The litter removed from the Middle Tisza at Kisköre Dam (Katona 2019) contains woody debris (9%), communal waste (12%), and other organic material (79%) (e.g., leaves, branches, and grass) (Figure 2), thus the ratio of the natural to anthropogenic litter is 88:12. Interestingly, the highest flood waves (between 2007 and 2017) were accompanied by the greatest volumes of riverine litter that was lifted (e.g., in 2010, 2015, and 2017) (Katona 2019), indicating the significant role of floods on litter transport.

## Material and Methods

**Figure 3.**
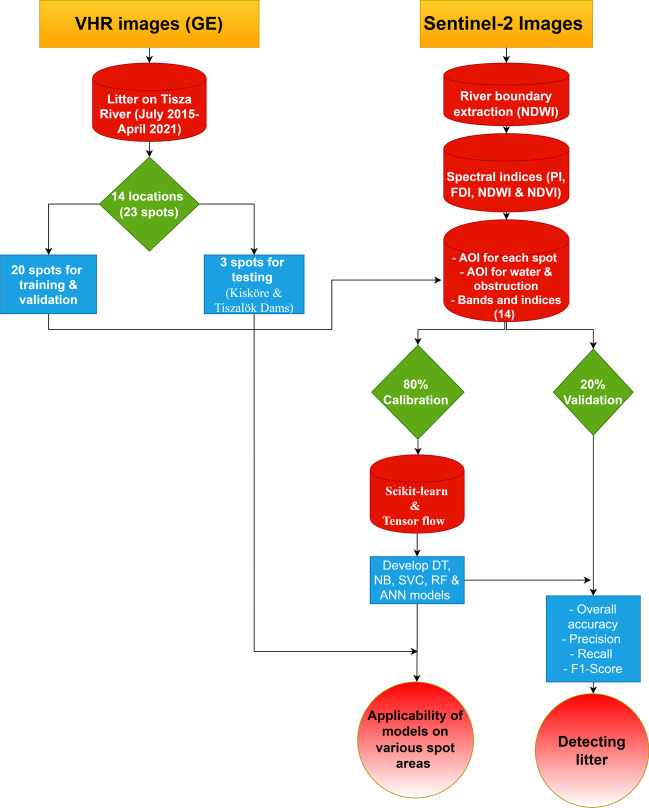
Flowchart summarizing the methodology of the study

The VHR images provided by the Google Earth database were utilized to identify litter spots along the Tisza River (Hungary), and they were employed to train, validate and test five ML algorithms classifying Sentinel-2 images (Figure 3). The best-performing model was employed to map the spatio-temporal distribution of riverine litter along a 175 km-long section of the Middle Tisza.

### Remote sensing data

#### Very High spatial Resolution (VHR) images

Sentinel-2 images could provide a reliable and recurring monitoring option for riverine litter as a result of their free availability, the high temporal resolution of 3-5 days, and their wide spectral range of 13 bands. However, their medium spatial resolution (10-60 m) increases the chances of false detection of riverine litter manually. In contrast, the VHR images from the Google Earth database could provide data about litter accumulation spots in rivers due to their high spatial resolution (< 1m), making it possible to recognize fine details. Therefore, the lower altitude orbiting sensors of the Google Earth database which provides VHR images (e.g., Airbus constellation: Pléiades Neo and Pléiades: 50 cm; Maxar constellation: Worldviews 3: 31cm; GeoEye: 41 cm) were employed to search for litter accumulation spots along the Tisza River, Hungary, between July 2015 and May 2021 (Figure 1A and B). Altogether 13 VHR images containing litter spots were employed including 3 images from Maxar and 10 images from Airbus constellations. Due to the limited number of available VHR images, historical Sentinel-2 images were also explored focusing on the identified spots in the VHR images to increase the dataset size. Besides, we concentrated mainly on the large and temporally static litter spots (e.g., upstream of hydraulic structures) to avoid the labeling errors of litter spots in Sentinel-2 images. During the labeling process, only pixels with reflectance anomalies were considered. All the identified litter spots in the VHR and Sentinel-2 images were used to train, validate, and test the ML algorithms.

#### Sentinel-2 images

Altogether 77 Sentinel 2A-B images were acquired from the Copernicus Open Access Hub (ESA 2021a) (2015-2021). Sixteen images were used to train, validate, and test the ML algorithms, while 61 images were used to reveal the spatio-temporal distribution of the litter along the Middle Tisza. The 16 images were obtained from the “T34UEU”, “T34TDT” and “T34TDS” tiles, as they covered almost the whole river in Hungary; moreover, they were selected to be as synchronous as possible with the acquisition dates of the VHR images (mean difference: 2.6 days). The 61 images were obtained from the “T34TDT” tile.

Most of the acquired images were in level-2A; however, older images (e.g., 2015-2016) were available in level-1C; thus, they were atmospherically corrected by the Sen2Core 255 processor in SNAP 8.0 software (ESA 2021b). All bands were resampled to 10 m using the nearest neighbor resampling method, where each pixel value in the new band is replaced by the value of its closest neighbor from the original band based on the Euclidean distance. Then, the Normalized Difference Water Index (NDWI) (McFeeters 1996) was applied to extract the bank lines. Three classes were identified: (a) accumulated litter spots, (b) water, and (c) man-made structures (dam, bridge, and ship). For each litter spot, an Area of Interest (AOI) was identified, and the reflectance values for each pixel were extracted; thus, 10 spectral reflectance values representing the bands; B2 (492 nm), B3 (559 nm), B4 (664 nm), B5 (704 nm), B6 (740 nm), B7 (782 nm), B8 (832 nm), B8a (864 nm), B11 (1613 nm), and B12 (2202 nm) were extracted. As the mean width of the Tisza is 164 m, the river is covered by ca. 16 pixels, which makes the labeling process of the historical Sentinel-2 images difficult at pixel scale. Therefore, only the pixels which showed significant reflectance anomalies were considered as litter, thus the pixels located on the peripheries of the large litter spots were discarded. For the water and obstruction classes, the same number of pixels as the litter class was identified.

The Plastic Index (PI), Floating Debris Index (FDI), NDWI, and Normalized Difference Vegetation Index (NDVI) (Rouse et al. 1974) in Equations 1-4 were integrated into the training process of the models. Thus, they and the spectral bands were considered as independent variables. The range of the PI (0-1) is consistent with the reflectance of the bands; however, the FDI, NDWI, and NDVI range between -1 and +1, therefore they were normalized using the Min-Max Normalization technique (Yu et al. 2009) in Equation 5.

1 $$\textrm{PI}=\frac{R_{NIR}}{R_{NIR}-{R}_{RED}}$$

2 $$\textrm{FDI}={R}_{NIR}-\left[{R}_{RE2}+\left({R}_{SWIR1}-{R}_{RE2}\right)\times \frac{\left({\lambda}_{NIR}-{\lambda}_{RE D}\right)}{\left({\lambda}_{SWIR1}-{\lambda}_{RE D}\right)}\times 10\right]$$

3 $$\textrm{NDWI}=\frac{R_{GREEN}-{R}_{NIR}}{R_{GREEN}+{R}_{NIR}}$$

4 $$\textrm{NDVI}=\frac{R_{NIR}-{R}_{RED}}{R_{NIR}+{R}_{RED}}$$

5 $$X^\backprime=\frac{X-\min(X)}{\max(X)-\min(X)}$$

Where *R*_*NIR*_, *R*_*RED*_, *R*_*RE*2_, *R*_*SWIR*1_ *and R*_*GREEN*_ are the reflectance of the near-infrared (NIR), red (RED), red edge 2 (RE2), shortwave infrared 1 (SWIR1) and green (GREEN) bands of the Sentinel-2 images; *λ*_*NIR*_, *λ*_*RED*_, *and λ*_*SWIR*1_ are the central wavelength of the NIR, RED, and SWIR1 bands; *X* is the original pixel value, *X`* is the normalized pixel value, and *min*(*X*) and *max*(*X*) are the minimum and maximum values of *X*.

### Training, validation, and testing of the applied algorithms

Our goal was to detect only riverine litter and differentiate it from the background (water and obstructions), which were considered less important classes in our study. To achieve this, the three-pixel classes (litter spot, water, and obstruction) were transformed into binary classes: litter (litter spots) and non-litter (water and obstruction). This conversion would simplify and accelerate the training process while improving performance with algorithms specifically designed for binary classification, such as SVC (Chih-Wei & Chih-Jen 2002). All pixels were divided into training, validation, and testing datasets. Three litter spots with various areas (and their surrounding ≈ 1.0 km of the river) were selected for testing, while the rest of the data were used to train and validate the algorithms. The area of a pixel is 100 m^2^, therefore, we selected one small litter spot (≤1 pixel), another medium spot (2-4 pixels), and one large spot (≥4 pixels) for testing the derived algorithms. The selected small and medium spots were located upstream of the Tiszalök Dam, while the large spot was upstream of the Kisköre Dam (Figure 1). The estimated area of the three spots by the derived models was compared with the area determined based on the VHR image. On the other hand, the rest of the pixels (1974 pixels) were used for training and validation. The Python scikit-learn library was used to split these pixels randomly to 80% for training and 20% for validation.

Five supervised ML algorithms, such as Decision Tree (DT), Naïve Bays (NB), Support Vector Classifier (SVC), Random Forest (RF), and Artificial Neural Network (ANN), were applied to build binary classification models to identify litter spots. All applied settings and parameters were chosen after fine-tuning to get the best available classification results. The fine-tuning was performed manually by defining a bounded domain for every hyperparameter and selecting random values until reaching the best classification accuracy. The SHapley Additive exPlanations (SHAP) (Lundberg &Lee 2017) was used to explain the contribution of each feature (i.e., bands and spectral indices) in the overall accuracy of the five models. The contribution of each feature to the overall accuracy of the model is calculated by assuming the accuracy in the case of all features included on one hand, and all but the specific feature included on the other hand. Thus, the contribution of this feature could be identified. The contribution of each feature is represented in a SHAP value, which is defined as the average of the marginal contributions across all permutations. The summary plot was adopted to represent the results of the SHAP analysis which shows not only the features sorted in descending order but also how they are affecting either positively or negatively. As this study applied five machine learning algorithms (i.e., DT, NB, SVC, RF, and ANN) with different structures, the three explainers (i.e., tree, kernel, and deep explainers) were applied. The tree explainer was used for the DT and RF models, the deep explainer with ANN, and the kernel explainer with the NB and SVC.

The Decision Tree (DT) is a non-parametric supervised ML algorithm. It splits the nodes on all entered features; then, the algorithm decides the further splits based on the homogeneity of the resulting sub-nodes. As several DT algorithms control the splitting of nodes (Rokach &Maimon 2005), in this study, the CART algorithm integrated into the scikit-learn library was applied to build the model. The “Gini” criterion was applied to measure the quality of the split; moreover, the “best” splitter method was applied.

The Naïve Bays (NB) is a probabilistic supervised classifier developed based on Bayes' theorem and Naïve assumption (Friedman et al. 1997). The scikit-learn library was applied to build an NB model to identify litter spots. The library has three NB algorithms; we applied the Gaussian NB, assuming that the features follow a normal distribution (Biermann et al. 2020). For each pixel, the algorithm calculated its probability to be litter or non-litter, based on the reflectance and the spectral indices values; afterward, the algorithm assigned this pixel to the highest probability class.

The Support Vector Machine (SVM) aims to draw a hyperplane in “n” dimensions, with the maximum margin separating the data into classes distinctly (Cortes &Vapnik 1995). The maximization of margin distance provides some sort of guarantee that the future predicted point would be classified accurately. The Support Vector Classifier (SVC) algorithm in the scikit-learn library was applied. The SVC has several kernels (i.e., linear, polynomial, sigmoid, and Radial Basis Function-RBF) used based on the nature of the designated data. These kernels were tested; finally, the RBF was applied, as it produced the highest accuracy. The regularization parameter (c) was set to 1000.

The Random Forest (RF) is based mainly on the decision tree algorithm (Breiman 2001). The input data are randomly divided into subsets through bagging or bootstrap aggregating to form small decision trees. Every decision tree contains a certain number of features, and it produces its decision based on these features and the selected input. The final decision of the RF is based on the average or majority voting of the resulting decisions from the subsets. The RF classifier in the scikit-learn library was applied. After testing, the accuracy did not improve significantly after 150 trees, thus the number of trees was set to 150.

The Artificial Neural Network (ANN) consists of a set of cells (neurons) connected to each other (McCulloch and Pitts 1943). The ANN is organized into three layers (input, hidden, and output). The neurons are connected by randomly initiated weights, then, they were updated during the training process. The output of the weighted sum of inputs is controlled by the activation function, then it is fed to the subsequent neuron. We applied the sequential model in Tensor Flow`s Keras API to build the litter model. The constructed model has one input layer consisting of 14 neurons, three hidden layers with 14, 12, and 8 neurons, and one output layer with one neuron. The ReLU was selected as the activation function for the hidden layers, while the sigmoid function was applied for the output layer. A dropout layer with a rate of 0.5 was added to avoid overfitting. The “binary cross-entropy” loss function and the “adam” optimizer functions were applied. The batch size was set to 32 and epochs to 400.

To validate the derived ML models, 20% of pixels were used. The classification report and the Cohen kappa score metrics in the scikit-learn library were applied to calculate the overall accuracy, precision, recall, F1-score, and Cohen kappa score (K-hat) for the validation and testing datasets (Equations 6-10; Congalton 1991). These metrics are calculated based on four sets of scores: True Positive (TP), True Negative (TN), False Positive (FP), and False Negative (FN). Additionally, random classifications of the same size as the testing dataset were generated and compared to the accuracy of the models to ensure that they were thoroughly trained. The overall accuracy, precision, recall, and f1-score range from 0 to 1 with the best results being closer to 1; meanwhile, the Cohen kappa score (K-hat) ranges from -1 to 1 with values close to 1 indicating strong agreement between the predicted and ground truth data.

6 $$\textrm{Overall}\ \textrm{accuracy}=\frac{TP+ TN}{TP+ TN+ FP+ FN}$$

7 $$\mathrm{Precision}\;\left(\mathrm{user}{'}\mathrm s\;\mathrm{accuracy}\right)=\frac{TP}{TP+FP}$$

8 $$\mathrm{Recall}\ \left({\mathrm{producer^{'}\mathrm{s}}\;}\mathrm{accuracy}\right)=\frac{TP}{TP+ FN}$$

9 $$\textrm{F}1-\textrm{score}=2\ast \frac{Recall\ast Precision}{Recall+ Precision}$$

10 $$\textrm{K}-\textrm{hat}=\frac{\left( observed\ agreement- expected\ agreement\right)}{\left(1- expected\ agreement\right)}$$

$$\textrm{where}:\textrm{observed}\ \textrm{agreement}=\frac{\left( TP+ TN\right)}{\left( TP+ TN+ FP+ FN\right)}$$

$$\textrm{and}\ \textrm{expected}\ \textrm{agreement}=\frac{\left[\left( TP+ FP\right)\ast \left( TP+ FN\right)+\left( TN+ FN\right)\ast \left( TN+ FP\right)\right]}{{\left( TP+ TN+ FP+ FN\right)}^2}$$

The spatio-temporal dynamism of the litter spots was analyzed in the Middle Tisza by applying the best-derived algorithm to all available images (61 Sentinel-2 images: 2015-2021). The seasonal Mann-Kendall trend analysis test (Kendall 1975, Mann 1945) was applied to the time series of the area of litter spot No. 8 (Figure 1) to reveal the temporal trend in litter accumulation. As the river behaves differently during the low and high stages, the seasonal Mann-Kendall test was employed considering two seasons: flood (March-April & June-July) and non-flood (August-February) seasons.

## Results

### Litter spots and spectral signature

Based on the visual interpretation of the VHR images, 14 litter spots were detected along the Hungarian section of the Tisza River (Table 1., Figure 1). Some were identified only once at most locations; however, upstream of hydraulic structures, the accumulation spots appeared repeatedly (e.g., at the Kisköre Dam, Figure 1). Within the investigated period (2015-2021) the most spots appeared in 2020. Within a year, usually they appear in March, June, and August. The size of the spots ranges from 25 m^2^ (<1 pixel) to 9,600 m^2^ (96 pixels).

**Table 1.** Characteristics of the identified litter spots (Figure 1) acquired by the Very High Resolution (VHR) images and the Sentinel-2 images. The three spots used for testing are indicated in bold

ID	Latitude	Longitude	Date (VHR)	Date (S2)	Area (m^2^) (pixels)
1	48°22'26"N	22°15'24"E	31/3/2019	23/3/2019	88 (<1)
2	48°23'13"N	22°08'02"E	31/3/2019	23/3/2019	75 (<1)
3/A	48°15'59"N	21°57'36"E	-	23/3/2019	140 (≈1)
3/B			5/4/2019	2/4/2019	130 (≈1)
3/C			28/6/2019	26/6/2019	130 (≈1)
4	48°11'20"N	21°43'38"E	3/6/2017	4/6/2017	267 (≈3)
**5/A**	**48°01'33"N**	**21°18'30"E**	**1/6/2019**	**1/6/2019**	**345 (≈3)**
**5/B**			**1/6/2019**	**1/6/2019**	**25 (<1)**
6	47°52'06"N	21°03'58"E	20/3/2020	20/3/2020	40 (<1)
7	47°49'17"N	21°01'17"E	20/3/2020	20/3/2020	520 (≈5)
8/A	**47°29'37.4"N**	**20°30'53.8"E**	27/6/2017	24/6/2017	3405 (≈34)
8/B			-	3/8/2017	4000 (40)
8/C			-	24/4/2020	4300 (43)
8/D			-	28/7/2020	9500 (95)
**8/E**			**1/8/2020**	**2/8/2020**	**9474 (≈95)**
8/F			-	12/8/2020	9000 (90)
8/G			-	22/8/2020	9600 (96)
9	47°22'14.54"N	20°26'7.66"E	29/7/2020	28/7/2020	204 (≈2)
10	47°15'11.18"N	20°23'51.50"E	9/8/2020	12/8/2020	220 (≈2)
11	47° 2'35.30"N	20°16'20.18"E	14/8/2017	8/8/2017	266 (≈3)
12	47° 2'35.30"N	20°16'20.18"E	9/8/2020	12/8/2020	181 (≈2)
13	46°57'1.78"N	20° 6'10.71"E	2/4/2020	4/4/2020	67 (<1)
14	46°14'40"N	20°09'02"E	3/3/2021	7/3/2021	90 (<1)

**Figure 4.**
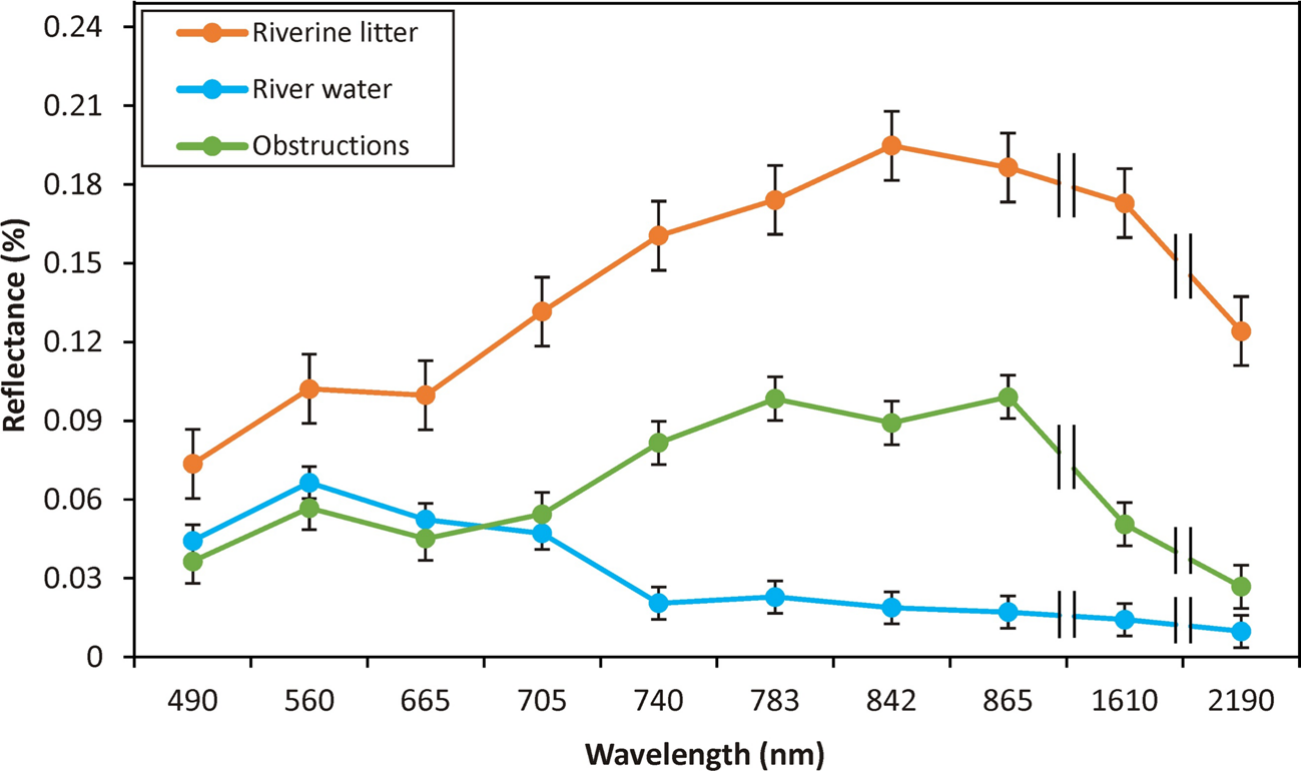
Average reflectance of the riverine litter, obstructions, and water of the identified pixels on the Sentinel-2 images

The spectral signatures of litter, water, and obstruction based on the Sentinel-2 bands were depicted (Figure 4). Generally, the litter showed the highest reflectance along the spectrum. The water pixels showed high reflectance in the visible bands (especially in green: 560 nm); however, they dropped significantly in the near-infrared (NIR) and shortwave-infrared (SWIR) bands (705-2190 nm). The spectral signature of the litter was very similar to the obstruction, as they have high reflectance at the SWIR bands (1610 and 2190 nm) and significant reflectance at the NIR bands (740-865 nm); however, they showed low reflectance in the visible bands, especially in blue (490 nm) and red (665 nm). Thus, the NIR and SWIR bands are the most suitable to discriminate the litter and obstructions from water. The discrimination between litter and obstructions is challenging; however, they could be differentiated by the 842 nm and 1610 nm wavelengths, where the reflectance of the obstruction pixels slightly drops, whereas the reflectance of the litter pixels reaches its peak.

### Validation of algorithms

The SVC achieved the highest classification metrics, while the NB achieved the lowest (Table 2.). The performance of the DT, SVC, RF, and ANN on the validation dataset was remarkable and comparable; for instance, the overall accuracy ranged from 0.93 (DT) to 0.96 (SCV). Due to the tradeoff between precision and recall, it is difficult to produce a very high precision and recall algorithm together. The ANN gave the best precision (0.98), but its recall was 0.89. The SVC gave the best recall (0.99), but with lower precision (0.9). The F1-score and K-hat demonstrated a similar pattern as accuracy in terms of the performance ordering of the five models. The accuracy of the random classification was significantly lower than its counterpart in the models, indicating that the models effectively learned the underlying patterns in the features and did not provide random predictions. Although the metrics of the five tested algorithms are relatively high, it is worth noting that the validation dataset size is relatively small due to a shortage of riverine litter data; hence, the models' performance may vary with a larger dataset. Therefore, the next section tested the performance of all models to determine their generalization capability and the smallest size of litter spots that could be detected by each model, utilizing three litter spots of varying sizes (small, medium, and large spots).

**Table 2.** Validation metrics for the five applied algorithms based on the validation dataset

Algorithm	Overall accuracy	Precision	Recall	F1-score	K-hat	Random classification
						Overall accuracy
Decision Tree (DT)	0.93	0.89	0.92	0.90	0.77	0.5
Naïve Bayes (NB)	0.89	0.86	0.80	0.83	0.58	0.55
Support Vector Classification (SVC)	**0.96**	0.9	**0.99**	**0.94**	**0.85**	**0.42**
Random Forest (RF)	0.94	0.88	0.95	0.91	0.84	0.45
Artificial Neural Network (ANN)	0.95	**0.98**	0.89	0.93	0.82	0.43

The text and values bold emphasized show other context of the data, or these are higher numbers as the results regarding to the other result number

**Figure 5.**
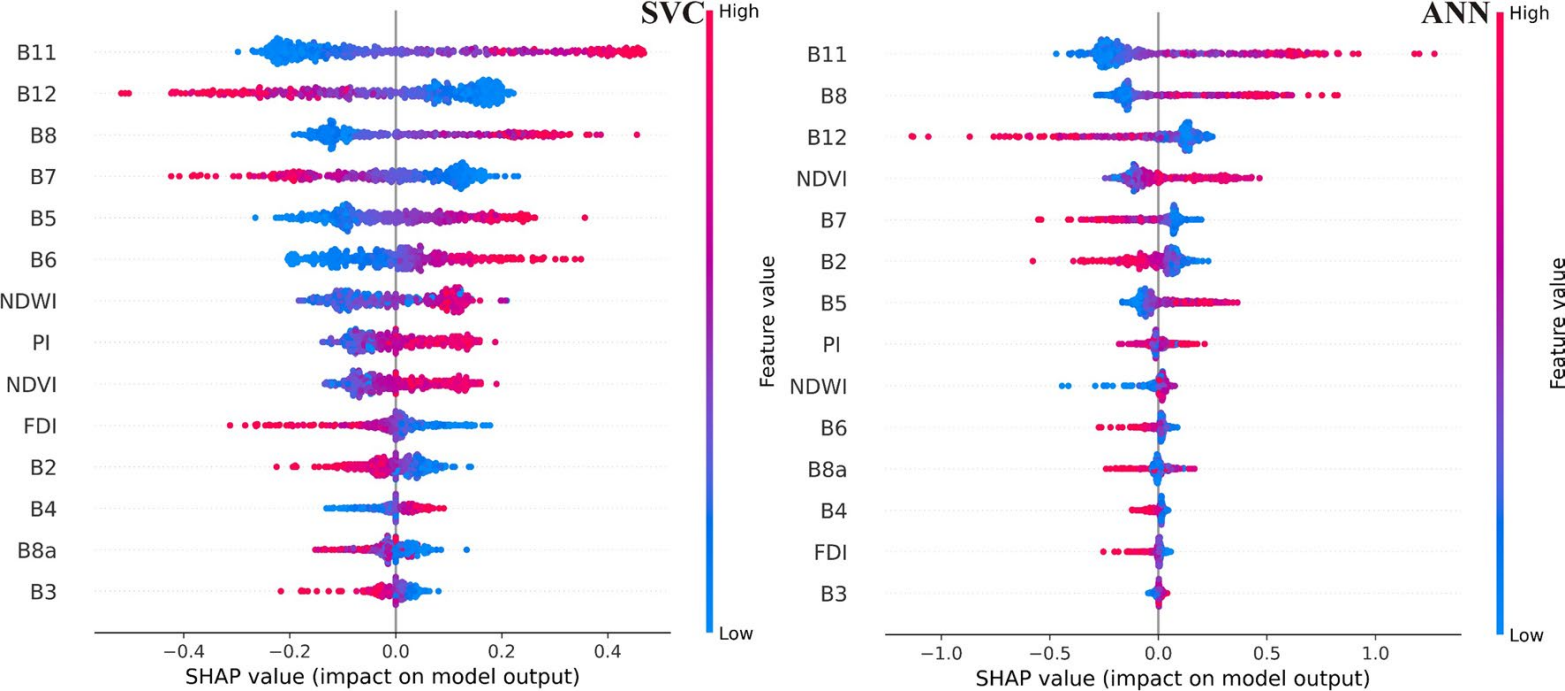
On the SHAP plots the importance of each input feature is expressed. Here the best-performing models (SVC and ANN) are presented

To understand the contribution of each independent feature (i.e., bands and spectral index) in the prediction process, a SHAP summary plot was produced (Figure 5). On the vertical axis, the various features are sorted in descending order according to their contribution to the model predictions, while the SHAP sign on the horizontal axis refers to the affection way of every independent feature in the model prediction. Based on the SHAP plots of the five models the SWIR (B11 and B12), NIR (B7 and B8), and spectral indices (PI, NDWI, and NDVI) were the most important features influencing the models. The most important contributing features for the DT were B11, PI, NDVI, and, B12; for the NB were B11, B12, B8, and NDWI; for the SVC were B11, B12, B8, and B7; for the RF were PI, B11, NDVI, and NDWI; and for the ANN were B11, B8, B12, and NDVI. The B11 was the highest contributing independent feature for four models (except for RF), followed by B12 and B8. The spectral indices, especially the PI, NDWI, and NDVI showed also high contributions, while the FDI was the least contributing index in all models.

### Testing the algorithms

**Figure 6.**
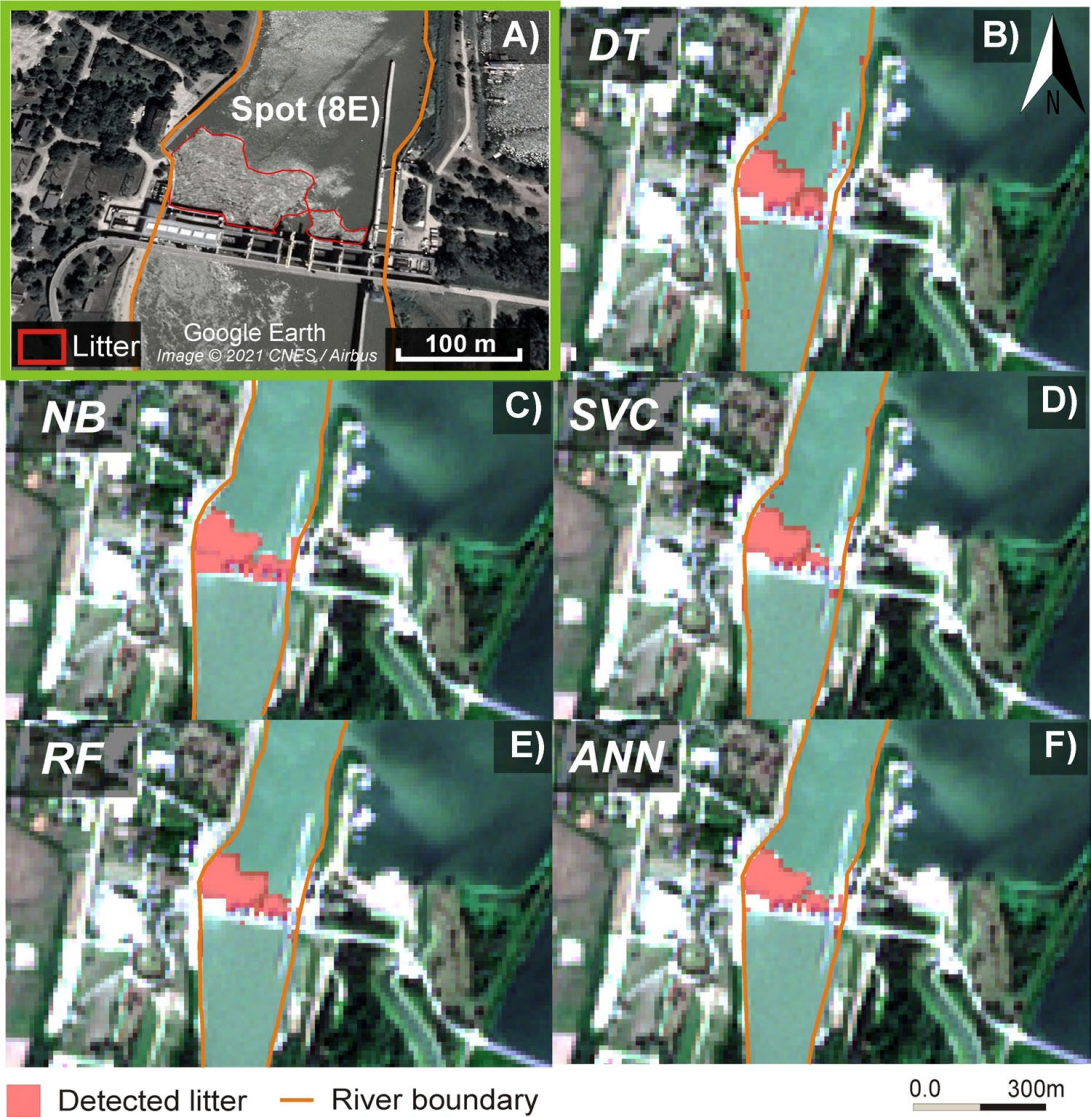
**A**) A large litter spot upstream of the Kisköre Dam was identified on the VHR image (12/08/2020). The area of the same litter spot was identified differently by the algorithms. **B**: Decision Tree (DT), **C**: Naïve Bayes (NB), **D**: Support Vector Classification (SVC), **E**: Random Forest (RF), and **F**: Artificial Neural Network (ANN). Background: Sentinel-2 (RGB 432)

**Figure 7.**
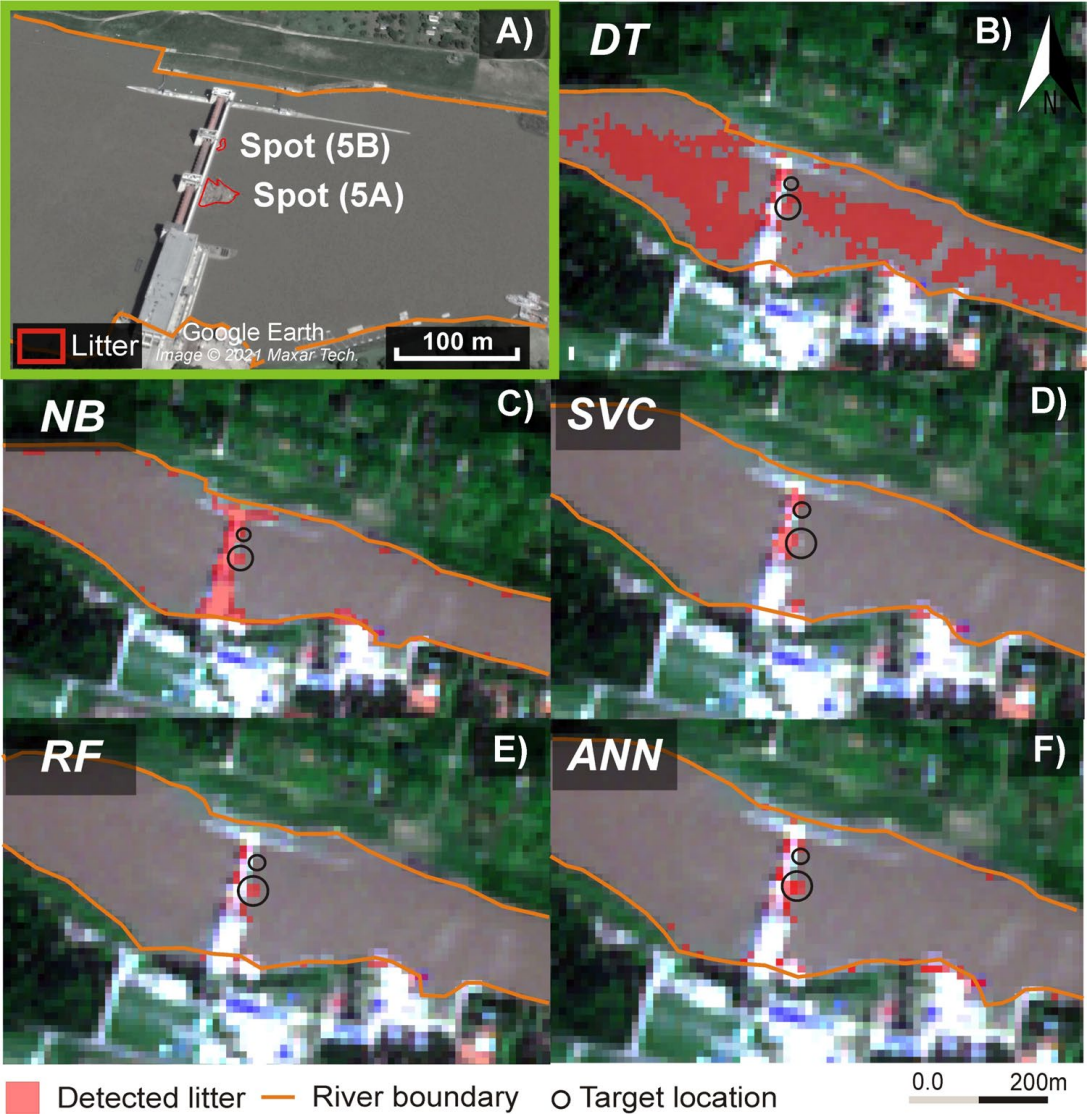
**A**) The area of the medium and small litter spots upstream of the Tiszalök Dam on the VHR image (01/06/2019). The area of the same litter spot was identified variously by the algorithms. **B**: Decision Tree (DT), **C**: Naïve Bayes (NB), **D**: Support Vector Classification (SVC), **E**: Random Forest (RF), and **F**: Artificial Neural Network (ANN). Background: Sentinel-2 (RGB 432)

To assess the generalization capability of the developed models, they were tested on a larger dataset (Table 3., Figure 6-7). Besides, the actual area of the three testing litter spots ( Figure 1, Table 1.) was compared to their estimated areas by the five models (Table 4, Figure 6-7). The developed models exhibited varying degrees of generalization capability, ranging from a medium in the case of SVC, RF, and ANN (F1-score range: 0.62-0.69) to poor in the case of DT and NB (F1-score: 0.45-0.48). The models demonstrated high recall (0.8-0.98) in identifying litter pixels, especially the large spot; however, they struggled to avoid false detection of non-litter pixels, particularly for DT and NB, resulting in a precision range of 0.3-0.56. It is worth noting that the overall accuracy (0.89-0.97) of the models was relatively higher than F1-score (0.45-0.69) due to their high performance in the majority class (water and obstructions), which overshadowed their poor-medium performance in the minority class (litter).

**Table 3.** Evaluation metrics for the five developed models based on the testing dataset

Algorithm	Overall accuracy	Precision	Recall	F1-score	K-hat
Decision Tree (DT)	0.89	0.30	0.9	0.45	0.44
Naïve Bayes (NB)	0.92	0.32	0.98	0.48	0.48
Support Vector Classification (SVC)	0.97	0.51	0.8	0.62	0.62
Random Forest (RF)	0.96	0.56	0.9	0.69	0.68
Artificial Neural Network (ANN)	0.97	0.51	0.8	0.62	0.62

**Table 4:** The applied models resulted in different areas (m^2^, pixels) for the same selected litter spots with various sizes

Method	Large spot m^2^ (pixels)	Medium spot m^2^ (pixels)	Small spot m^2^ (pixels)
	Kisköre Dam (02/08/2020)	Tiszalök Dam (01/06/2019)	
**VHR images from GE (Reference data)**	**9474 (95)**	**345 (4)**	**25 (<1)**
Decision tree (DT)	13400 (134)	400 (4)	0.0 (0)
Naïve Bays (NB)	10100 (101)	400 (4)	100 (1)
Support Vector Classifier (SVC)	11500 (115)	200 (2)	0.0 (0)
Random Forest (RF)	12000 (120)	400 (4)	0.0 (0)
Artificial Neural Network (ANN)	11200 (112)	400 (4)	0.0 (0)

The text and values bold emphasized show other context of the data, or these are higher numbers as the results regarding to the other result number

The largest spot upstream of the Kisköre Dam (9474 m^2^; 95 pixels) was detected by all models with good accuracy; however, most of them slightly overestimated its area (Figure 6, Table 4). The DT and RF provided the greatest over estimated area, 3926 m^2^ (ca. 40 pixels) and 2526 m^2^ (26 pixels), respectively. However, these algorithms correctly classified most of the dam body and water as non-litter targets. The NB gave also a good areal estimation, as it misclassified only an area of 626 m^2^ (≈6 pixels) as litter; but it misclassified almost the entire dam as litter. The ANN and SVC provided the best detection accuracy, as the ANN misclassified only 1726 m^2^ (≈18 pixels), and the SVC misclassified 2026 m^2^ (≈21 pixels); besides, they correctly classified the dam and water.

The algorithms identified the medium spot (345 m^2^) with various accuracies, but only the NB could identify the small spot (25 m^2^, <1 pixel), though, with low accuracy (Figure 7, Table 4). Although the DT identified the four pixels of the medium spot, it misclassified a significant area of the water as litter, but it correctly classified most of the dam as non-litter. The NB was the only algorithm that detected both medium and small spots. However, it also misclassified the dam as litter. Despite the SVC having the highest accuracy and F1-score, it underestimated the area of the medium targets (2 pixels only were identified); however, its advantage is the low misclassification of water and dam pixels. The RF and ANN had similar results, as both detected the four pixels of the medium spot, but none of them detected the small spot. Both had a very low number of misclassified water and dam pixels; therefore, they could be considered the best estimators for medium spots.

### Spatiotemporal dynamism of litter spots

**Figure 8.**
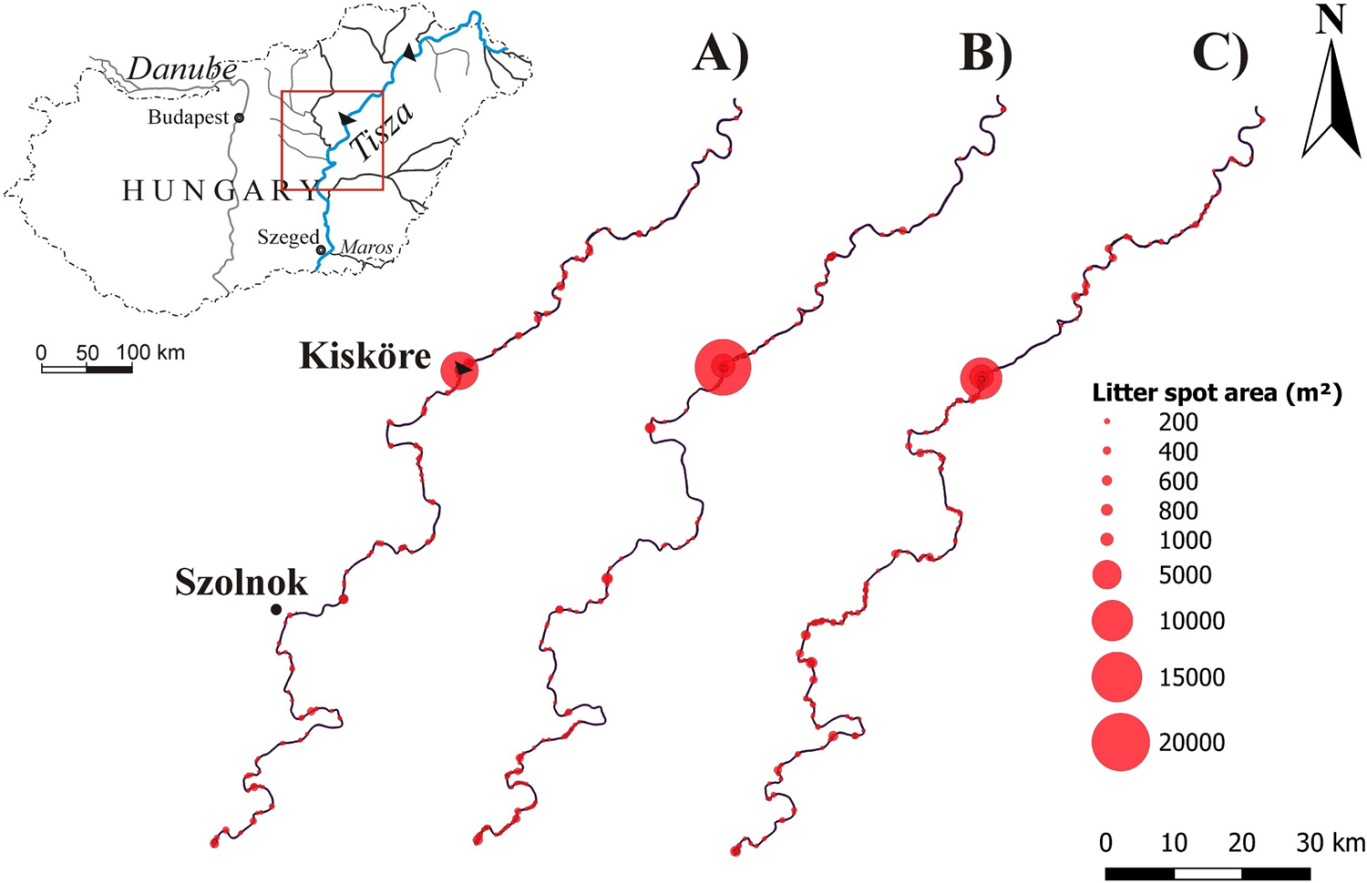
The identified litter spots along the 175 km-long section of the Middle Tisza, by applying the Support Vector Classifier (SVC) on Sentinel-2 images. **A**) bankfull flood (31/03/2019); **B**) small flood (09/06/2019); and **C**) low stage (22/09/2019)

The 175 km-long section of the Middle Tisza was analyzed in detail, by applying the SVC on Sentinel-2 images (2015-2021). The spatial distribution of litter spots during subsequent hydrological situations (e.g., bankfull flood, small flood, and low stage) were depicted as examples (Figure 8). The largest litter spot (2300-28,000 m^2^ or 23-288 pixels) developed upstream of the Kisköre Dam, though several small to medium spots were identified along the reach. Their total area varied between 36,900 and 99,000 m^2^ (369-990 pixels), and most of them were along the riverbanks, consistently with the identified spots on VHR images. However, the density of these spots was greater than what was observed in the VHR images. Probably because some pixels were misclassified as litter due to the shadow of trees and banks, or the bent and fallen trees were misclassified as litter. Some spots were observed around docks; but no accumulation was detected at bridge piers, consistently with the VHR images. The density of the identified litter spots was greater downstream of the Kisköre Dam (1.2-1.6 spot/km) than upstream of it (0.85-1 spot/km) during all presented hydrological situations (Figure 8). As the litter from the upstream section is trapped by the dam, probably the litter downstream originated from the banks. Litter spots were rarely identified along straight or low sinuosity sections, as they usually were found at meanders with retreating banks and falling trees.

**Figure 9.**
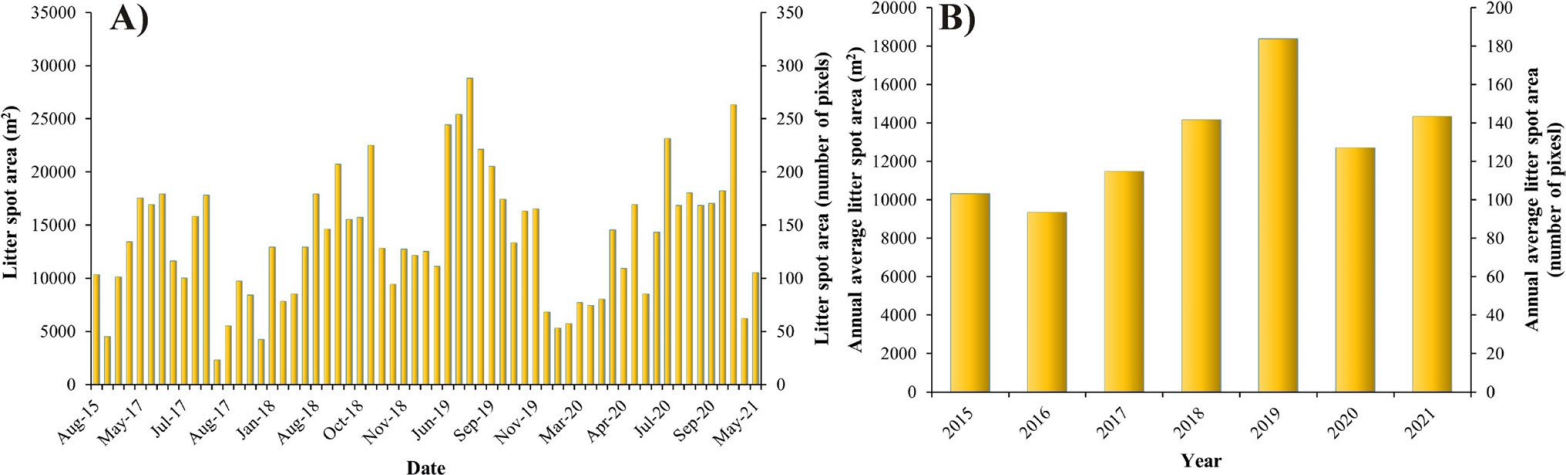
**A**) Temporal changes in the area of the largest litter spot at the Kisköre Dam applying the Support Vector Classifier (SVC) on 61 Sentinel-2 images (2015-2021). **B**) Annual average area of the litter spots

The location and size of the litter spots changed temporally among the subsequent hydrological situations (Figure 8). The area of the litter spot at Kisköre Dam after small flood-waves was greater than after the bankfull spring floods (Figure 8A, B), referring to the importance of gradual fluxes. During low stages, the area of this litter spot decreased (Figure 8C) as it was lifted by the authorities. Focusing on the litter spot upstream of the Kisköre Dam, the mean area almost doubled between 2015 (10,300 m^2^ or 103 pixels) and 2019 (18,367 m^2^ or 184 pixels) (Figure 9), but later it dropped by 30% in 2020; however, a slight increase (12%) was observed in 2021. An overall increasing trend was confirmed by the seasonal Mann-Kendall test (*p*-value=0.001; Mann-Kendall Statistic S=120; Kendall's Tau=1). This increasing area could be explained by the lack of large floods.

## Discussion

The five tested algorithms showed promising results in terms of riverine litter detection, especially the SVC and ANN as they can deal with continuous datasets, non-linear, and high dimensional classification problems. The presented binary nature of the classification problem also gave an advantage to SVC over DT and RF which are commonly used with multiclass problems. The SVC usually predicts the binary classes with high accuracies due to the formation of hyperplane and support vectors which separate the two classes distinctly. The NB gave the least prediction accuracy, as the bands and spectral indices used for prediction are correlated, thus the Bayes` assumption of independent condition was not satisfied. As the obstruction and water pixels were combined into one class the classification dataset became imbalanced; however, the imbalance ratio (non-litter: litter class=67:33) was considered as low, thus it did not affect the potential of the ML algorithms to detect the minority class (Zou et al. 2016).

The classification metrics in our study were comparable to the literature. For instance, Biermann et al. (2020) detected macro-plastics in marine litter using the NB classifier with an overall accuracy of 0.86, which is very close to our classification accuracy by the NB (0.89). However, it is worth noting that they tackled a multiclass problem, which might explain the slightly lower classification accuracy than ours. They also indicated that their main challenge stems from the limited availability of images depicting macro-plastics, leading to occasions where the classifier confuses macro-plastics with either seawater and/or sea foam. Our study also encountered similar misclassification issues with the NB classifier, particularly in regard to distinguishing between riverine litter and obstructions. The ANN detected the riverine litter in the Drina River, Bosnia and Herzegovina with an overall accuracy of 0.97 (Jakovljević et al. 2019), which is slightly higher than our classification accuracy by the ANN (0.95). However, the authors reported that the developed ANN model often underestimates the presence of litter, due to the misclassification of mixed pixels (pixels covered by both water and litter) or pixels covered by water as a non-litter class. Besides, the presence of optically active water constituents, mainly suspended sediment, might also affect the classification accuracy; however, in our study, water pixels were labeled under various hydrological conditions, covering a wide range of suspended sediment concentrations to overcome this issue.

Based on the SHAP summary plots, the B11, B12, and B8 bands highly contributed to all models, thus they could be employed to produce a litter spectral index in future studies. This could be interpreted by the spectral signature graph (Figure 4). The water has very low reflectance at these wavelengths, contrary to litter, thus they could be separated easily. The spectral signature of obstructions was very similar to the litter, therefore discrimination between them is challenging, but at these bands, the obstruction signatures are slightly to significantly lower than litter signatures. The integration of the various indices (especially PI, NDWI, and NDVI) in litter detection models was very useful, as they enhance the spectral feature of litter by minimizing the effects of illumination.

The tests on litter spot-sizes demonstrated that the classification of the SVC, ANN, and RF outweigh the DT and NB. Although the DT and NB models produced relatively high classification metrics in Table 2., they performed poorly during testing. This might be attributed to the limited validation dataset (395 pixels) compared to the more extensive and diverse testing dataset, which includes varying hydrological conditions and litter sizes. The main disadvantage of the DT and NB was the misclassification of water and obstructions as litter respectively. Although the imbalance ratio of the dataset used in this study has a slight impact on the applied algorithms, their impact on simple algorithm e.g., DT could be larger. Therefore, the DT tends to classify litter pixels (minority class) as non-litter to achieve the highest possible accuracy. The spectral signature of obstructions seems to be very close to litter, thus the NB failed to distinguish litter from the obstruction. Turbidity affects the spectral signature of water, especially in the red band, therefore the water pixels used for training and validation were collected at different hydrological conditions considering low and high suspended sediment concentrations. Therefore, most algorithms correctly classified water pixels except the DT which misclassified high turbidity water as litter.

The large and medium-sized litter spots were detected by the five algorithms correctly; however, the identification of small litter spots was challenging for almost all algorithms except for the NB. However, as the NB has problems separating obstructions and litter, this sub-pixel size detection is not certain. The good performance of the algorithms in identifying large and medium spots could be explained by that large number of litter pixels have more effective reflectance into the sensor; meanwhile, the area of the small litter spot is not large enough to affect the reflectance. Similar results were reached by Topouzelis et al. (2019), who concluded that a spot with an area of 10×10 m is an essential prerequisite to be detected on Sentinel-2 images.

Remote sensing of litter is always associated with the appearance of spatial reflectance anomaly (i.e., the litter pixel arises from the background pixels by its blinking reflectance). The discrimination between the various litter materials is related to the similarity and dissimilarity of their spectral signature, which depends on the Signal to Noise Ratio (SNR) of the sensor and band settings. The Multi-Spectral Imaging (MSI) sensor of Sentinel-2 has 13 spectral bands covering a wide range of the spectrum (including the NIR and SWIR) offering a good chance for litter detection. In addition, the very short revisit time of Sentinel-2 (3-5 days) could also be employed to monitor the temporal change of litter. On the other hand, the relatively low SNR (mean of all bands=116.5) and the difference in the spatial resolution of the bands (10 to 60 m) make the sub-pixel scale identification challenging. Although Hu (2021) reported that the minimum percentage coverage of a pixel for detection (based on a single band) or discrimination (based on combined bands) for the MSI sensor are 0.8% and 1% respectively, these thresholds did not apply to our study. As these values were calculated assuming that the image noise originates only from the sensor, while practically it could be affected by other sensor`s artifacts (e.g., pepper noise and hardware parallax impacts in push-broom). However, the sub-pixel detection of riverine litter needs further research, as the percentage of litter pixels having an area of less than one pixel in our training and validation dataset was just 1%, thus it was significantly underrepresented. Therefore, the ML models tend to misclassify them as non-litter, as their reflectance could be very close to water pixels.

The spatio-temporal distribution of riverine litter is very complex due to several factors affecting its transport. Along the Tisza, the largest and most persistent litter spots were observed upstream of dams. It agrees with studies that investigated plastic transport in rivers (Zhang et al. 2015). Besides, some litter spots were observed along the riverbanks, as there the flow velocity is low, and the natural (e.g., riparian trees) and artificial (e.g., docks) obstructions can trap debris. Extreme events (e.g., stormy winds, heavy rains, floods) control the temporal input of the litter in rivers, as they (re)mobilize the litter in the fluvial system. Usually, a high litter transport rate was observed during floods (Van Emmerik et al. 2019), similar to our observations in the Tisza River. However, we noticed a time-lag between the flood-waves and the formation of the largest spot area, which refers to the gradual transport and accumulation of litter during small flood-waves until reaching the greatest litter area at the end of the flood waves. For instance, the largest litter spot area in 2017 (178 pixels), 2018 (225 pixels), 2019 (288 pixels), and 2020 (231 pixels) were recorded in August, October, August, and July respectively, as these periods are featured with low stages after the completion of the flood waves.

This study is an initial attempt at the automatic detection of riverine litter using satellite images. Although, it provided promising results on the automatic detection of large and medium litter spots on Sentinel-2 images, the detection of small spots is still questionable. Though it was proved, that even a low abundance of litter spots could be detected, more research is needed considering larger datasets, with more representative small spots. Besides, a larger dataset would improve the models` performance and consequently their generalization capability. The Tisza River is considered as a medium-sized river (mean width: 164 m), thus it is covered by just 16 pixels of Sentinel-2 images, therefore applying this approach to finer spatial resolution images is suggested on medium-sized rivers to increase the chance of small litter detection.

## Conclusion

Organic litter on rivers is a natural phenomenon; however, since it is mixed with plastic waste it has become a worldwide problem. The catchment of the Tisza River (Central Europe) is shared by five countries, where the communal waste production is relatively high (average: 340 kg/capita) but its recycling is moderate or low. Therefore, a huge amount of plastic waste is mixed in the natural litter and transported downstream. For the successful prediction and mitigation of waste transported with the drifting litter, this study aimed to combine satellite images with ML algorithms to detect riverine litter and investigate its spatio-temporal dynamism.

The combination between Sentinel-2 images and machine learning algorithms (DT, NB, SVC, RF, and ANN), and the application of the Very High Resolution (VHR) images (Google Earth) proved their ability to support the identification of riverine litter. However, due to the 10 m spatial resolution of the Sentinel-2 images, most of the small litter spots (<100 m^2^) were missed, thus, the produced models are useful just to detect medium and large litter spots. However, special consideration should be given to the false detection of the non-litter pixel as litter, given that the models had moderate to poor precision. Based on the validation and testing results of the five tested algorithms, the SVC, RF, and ANN are highly recommended for further studies, as they showed the best performance. The proposed methodology was very helpful to analyze the spatio-temporal dynamism of the medium and large litter spots on a medium-sized river. However, applying the same methodology on finer spatial resolution satellite images (e.g., WorldView-3 and SPOT 5) would be very useful for investing the spatio-temporal dynamism of small litter spots.

On a deep river like the Tisza, litter spots accumulated upstream of dams and along riverbanks. They are mobilized during floods, but they are trapped during small flood-waves or low stages. We suggest that the litter removal campaigns should be held at the end of these periods when the litter spots are more likely to be accumulated at well-defined locations. Although promising results were obtained by the proposed methodology on a middle-sized Tisza River, probably better performance could be reached on larger rivers. Remote sensing with its large-scale coverage and frequent capturing can be employed as a good monitoring tool for the spatio-temporal distribution of riverine litter, understanding its sources, hotspots, transport routes, and sinks.

## Data Availability

Data will be made available on request. The implemented code in this study is available at https://github.com/AMohsenMetwaly/Macroplastic_RS_ML.
